# Re-Emergence of Crimean-Congo Hemorrhagic Fever Virus in Central Africa

**DOI:** 10.1371/journal.pntd.0001350

**Published:** 2011-10-11

**Authors:** Gilda Grard, Jan Felix Drexler, Joseph Fair, Jean-Jacques Muyembe, Nathan D. Wolfe, Christian Drosten, Eric M. Leroy

**Affiliations:** 1 Centre International de Recherches Médicales de Franceville (CIRMF), Franceville, Gabon; 2 Institute of Virology, University of Bonn Medical Centre, Bonn, Germany; 3 Global Viral Forecasting, San Francisco, California, United States of America; 4 Institut National de Recherche Biomédicale, Kinshasa, Democratic Republic of the Congo; 5 Department of Epidemiology, School of Public Health, University of California Los Angeles, Los Angeles, California, United States of America; 6 Institut de Recherche pour le Développement (IRD), UMR 224 (MIVEGEC), IRD/CNRS/UM1, Montpellier, France; Children's Hospital Oakland Research Institute, United States of America

## Abstract

**Background:**

Crimean-Congo hemorrhagic fever (CCHF) is a severe tick-borne disease well recognized through Europe and Asia where diagnostic tests and medical surveillance are available. However, it is largely neglected in Africa, especially in the tropical rainforest of Central Africa where only sporadic human cases have been reported and date back to more than 30 years. We describe here an isolated human case that occurred in the Democratic Republic of the Congo in 2008 and performed phylogenetic analysis to investigate whether it resulted from a regional re-emergence or from the introduction of a novel virus in the area.

**Methods and Findings:**

Near complete segment S and partial segment M sequences were characterized. Bayesian phylogenetic analysis and datation were performed to investigate the relationship between this new strain and viral strains from Africa, Europe and Asia. The new strain is phylogenetically close to the previously described regional genotype (II) that appears to be specific to Central Africa. Phylogenetic discrepancy between segment S and M suggested genetic exchange among local sublineages, which was dated back to 130–590 years before present.

**Conclusions:**

The phylogenetic analyses presented here suggest ongoing CCHF virus circulation in Central Africa for a long time despite the absence of reported human cases. Many infections have most probably been overlooked, due to the weakness of healthcare structures and the absence of available diagnostic procedure. However, despite the lack of accurate ecological data, the sporadic reporting of human cases could also be partly associated with a specific sylvatic cycle in Central Africa where deforestation may raise the risks of re-emergence. For these reasons, together with the high risk of nosocomial transmission, public health authorities' attention should be drawn to this etiological agent.

## Author Summary

Crimean-Congo hemorrhagic fever virus (CCHFV) is transmitted to humans through tick-bite or contact with infected blood or tissues from livestock, the main vertebrate hosts in a peri-domestic natural cycle. With numerous outbreaks, a high case fatality rate (3%–30%) and a high risk for nosocomial transmission, CCHFV became a public health concern in Europe and Asia. However virus surveillance in Africa is difficult due to the limited sanitary facilities. Especially, CCHFV occurrence in Central Africa is very poorly described and seems highly in contrast with the temperate to dry environments to which the virus is usually associated with. We described a single human infection that occurred in Democratic Republic of the Congo after nearly 50 years of absence. The phylogenetic analysis suggests that CCHFV enzootic circulation in the area is still ongoing despite the absence of notification, and thus reinforces the need for the medical workers and authorities to be aware of the outbreak risk. The source of infection seemed associated with a forest environment while no link with the usual agro-pastoral risk factors could be identified. More accurate ecological data about CCHFV enzootic cycle are required to assess the risk of emergence in developing countries subjected to deforestation.

## Introduction

Crimean-Congo hemorrhagic fever virus (CCHFV, family *Bunyaviridae*, genus *Nairovirus*) is a tick-borne virus. It causes severe illness throughout Africa, Asia, Southeast Europe and the Middle East, with case fatality rates ranging from 3% to 30%. Its worldwide distribution closely matches that of its main arthropod vector, ixodid ticks belonging to the genus *Hyalomma*. Human infection occurs through tick bites, contact with infected livestock, or nosocomial transmission. The CCHFV negative-stranded RNA genome is divided into a small (S), medium (M) and large (L) segment.

Previous phylogenetic analysis of the S segment clustered strains into 6 to 7 distinct phylogeographic groups: West Africa in group I, Central Africa (Uganda and Democratic Republic of Congo (DRC)) in group II, South Africa and West Africa in group III, Middle East and Asia (that may be split into 2 distinct groups Asia 1 and Asia 2 [4]) in group IV, Europe and Turkey in group V, and finally Greece in group VI [1]–[5]. However, some of these phylogenetic lineages include strains separated by large spatial distances (such as South Africa and West Africa) suggesting viral migration, most likely via migratory birds transporting infected ticks, or secondary introductions following importation of commercial livestock. Comparative phylogenetic analysis revealed, with a few exceptions, parallel clustering of the S and L segments, while M segment reassortment seems more frequent [1], [4]–[6].

During the last 60 years, CCHFV outbreaks have been described in Asia, the Middle East and the Balkans, where the virus has become endemic and caused several thousand human cases. During the last decade, CCHFV has caused human disease in previously unaffected countries (Turkey 2002, Iran 2003, Greece 2008, Georgia 2009) and has re-emerged in countries located southwest of the Russian Federation after an absence of nearly 30 years [7]. By contrast, fewer than 100 cases have been recorded in Africa [8], most of them in South Africa [9], [10]. In East and West Africa, enzootic CCHFV circulation has been shown by serological surveys of cattle and virus isolation from ticks since the 1970s [11], [12] but until the outbreaks in Mauritania in 2004 [13] and Sudan in 2008 [14], only sporadic human cases had been reported. In Central African Republic (CAR), limited serological evidences of CCHFV circulation in Zebu cattle has been provided [15] and three viral strains were isolated from ticks between 1973 and 1976, one of which lead to accidental infection of a laboratory worker [11]. Subsequent isolations from ticks were performed in the 80's [16] but no human case was reported. Despite the early identification of human CCHFV infection in DRC (Kisangani, 1956), CCHFV occurrence in Central Africa has not been much described and only sporadic human cases have been reported. One month after having isolated the first CCHFV strain (strain Congo 3011) in newborn mice, Dr. Courtois became infected and this was the last notified case in DRC, from which the strain Congo3010 was isolated [17], [18]. The virus was next identified in Uganda between 1958 and 1978. Fifteen CCHFV strains were isolated from febrile patients, of which nearly half were laboratory workers having handled infectious samples [11], [17], [18]. From the geographic information associated with the other patients, it can be inferred that CCHFV was present both in the Entebbe area and in the Arua district (previous West Nile district) located 350 km North, near the border of Sudan. Three CCHFV strains were also isolated from ticks and an early serological survey suggested cattle infection [11]. No other epidemiological or ecological information is available on CCHFV in Central Africa or its borders, and no further cases have been recorded.

In 2008, CIRMF (Centre International de Recherches Médicales de Franceville, Gabon) identified CCHFV in a serum sample received for etiological diagnosis of a case of hemorrhagic fever in DRC. This is the only identification of CCHV in DRC for more than 50 years. To determine whether it was due to introduction of a novel virus or to re-emergence of a local genotype, we determined the phylogenetic relationships between this virus (hereafter referred to as Beruwe-2008) and previously described isolates. Phylogenetic analysis showed that the Beruwe-2008 strain belonged to the genotype previously identified in this area and thus suggested that it had re-emerged. Local CCHFV persistence may have been supported by a sylvatic natural cycle specific to Central Africa, indicating that countries subject to major deforestation may note an increasing number of human infections.

## Methods

### Ethics statement

Laboratory investigations were performed subsequently to the WHO request for surveillance and early alert of hemorrhagic fever outbreak in Central Africa. Because of the emergency settings associated with the suspicion of such acute illnesses, no ethics committee approval or written consent was deemed necessary. The blood sample was taken by national healthcare workers of the Lubutu hospital where the patient came for medical care. He was informed that his blood sample will be further used for diagnostic investigation and gave his verbal consent. The patient described here is anonymous. The blood sample was next sent to the Institut National de Recherche Biomédicale (Kinshasa, DRC) and then to CIRMF upon WHO authorities. The study was approved by the scientific committee of CIRMF.

### Case description

**Figure 1:**
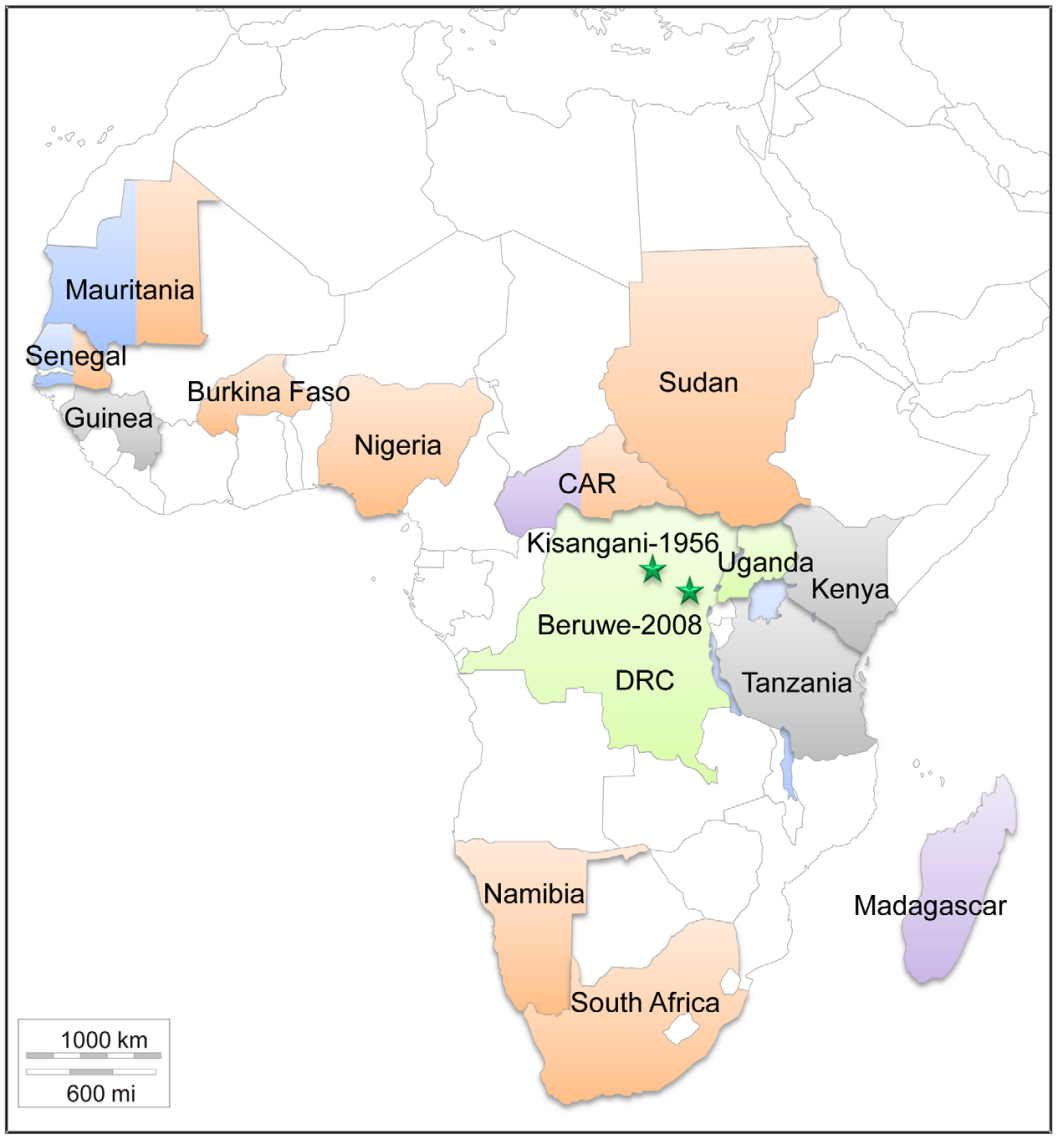
Geographic distribution of CCHFV in Africa. Genotypes I, II, III and IV are indicated in blue, green, orange and mauve, respectively. Undetermined genotypes are in grey. Stars represent human infections diagnosed in Democratic Republic of the Congo (DRC). CAR: Central African Republic.

The patient was a 26-year-old man living in Beruwe (Nord Kivu province) in DRC, 325 km from Kisangani (Figure 1). He became ill in the mining area where he worked. He complained of fever and headache on day 1 and developed moderate bloody diarrhea on day 2. Epistaxis, oral bleeding and hematuria occurred on day 3. He treated himself with ibuprofen and paracetamol during the first three days. On day 4 after onset he additionally took quinine and finally presented with severe asthenia and persistent bleeding to Lubutu hospital, where the serum sample was taken. At this stage the patient was subicteric, with bleeding at the venipuncture site, but had only low-grade fever (37.6°C). He declared no contact with wild animals during the previous three weeks but he had slept in the forest. No further information on his outcome was available.

### Molecular diagnosis

The patient's serum was manipulated in biosafety level 4 (BSL-4) conditions. The serum was first tested for Ebola and Marburg viruses. As results were negative, investigations were next performed for CCHFV. RNA was extracted with the QIAamp viral RNA mini kit (Qiagen, Courtaboeuf, France) according to manufacturer's instructions. Reverse transcription (RT) and real-time PCR amplification were performed with the High Capacity cDNA RT kit and Taqman universal PCR master mix (Applied Biosystems - Life Technologies Corporation, Carlsbad, California), and previously reported primers and probes [19]. Conventional one-step RT-PCR was performed with CCHFV primers as previously reported [20] and with SuperScript III one-step RT-PCR system and Platinum Taq DNA polymerase (Invitrogen -Life Technologies Corporation, Carlsbad, California). This yielded a 536-nucleotide fragment in the S segment, sequencing of which confirmed CCHFV identification.

### Genetic characterization

As viral isolation on Vero cells was unsuccessful, viral RNA was extracted from the patient's serum as described above, and was used for RT-PCR amplification with Platinum Taq DNA polymerase (Invitrogen). Primers were derived from nucleotide alignments (Table 1). Three overlapping PCR products allowed near-complete characterization of the S segment coding sequence (GenBank accession number HQ849545) and partial characterization of the M segment (GenBank accession number HQ849546). Amplification of the L segment was unsuccessful, being limited by the sample quantity.

**Table 1:** Primers used for CCHFV genetic characterization of the S and M segments.

Segment S		
Fragment 1 (590 bp)		
1^st^ round	CCHF-MU: TCTCAAAGATATCGTTGCC	CCHF-ER7: GAATTAGGGAAGCAACCAAG
Fragment 2 (550 bp)		
1^st^ round	CCHF-F2b: AAAGAGATGTTGTCAGACATGAT	CCHF-R2b: GTTTCTTTCCCCACTTCATTGG
2^nd^ round	CCHF-F3b: GAAGAAGGAACTTGATCCTCAA	CCHF-R2b
Fragment 3 (536 bp) [19]		
1^st^ round	CCHF-F2: TGGACACCTTCACAAACTC	CCHF-R3: GACAAATTCCCTGCACCA
Segment M		
Fragment 1 (280 bp)		
1^st^ round	CCHF-M1F: AATGCAATAGATGCTGAAATGCA	CCHF-M2R: TTGYTTGCYTC1AYRGTYGC
2^nd^ round	CCHF-M1F	CCHF-M1R: GAYTGRACTGG1GAYAWYGAAAC
Fragment 2 (420 bp)		
1^st^ round	CCHF-M2F: CAAGTRTCRGAGTCAACRGG	CCHF-M2R: TTGYTTGCYTC1AYRGTYGC
2^nd^ round	CCHF-M3F: TGGCTCTRAAGAGRAGYTGYTGGATRA	CCHF-M3R: TTRCARACRGCYAGCATRACATT
Fragment 3 (510 bp)		
1^st^ round	CCHF-M4F: GAGTC1CAYAAATGCTAYTGYAGTCT	CCHF-M4R: ACTGAACTCCAGCTAAGTGCTA
2^nd^ round	CCHF-M4F	CCHF-M5R: GTTGAYTGRACATTRATTGCYCCCCA

Sequences are reported in 5′-3′ orientation.

### Phylogenetic analysis

A total of 44 complete sequences for segment S and 38 complete sequences for segment M were retrieved from GenBank (Table S1 in online supporting information). Nucleotides were aligned according to the amino-acid profile using the Translation Align algorithm implemented in Geneious software [21]. Initial phylogenetic analyses were performed with MrBayes V3.1 [22], [23] using a GTR+gamma+invariant site substitution model for 4 million MCMC chain iterations sampled every 100 generations, corresponding to 40 000 trees (data not shown). Following confirmation of the tree topology from MrBayes, the tip-dated coding alignments were submitted to Bayesian inference of node ages by using BEAST V1.4.7 [24] under the assumption of a codon-based substitution model (SRD06) and an uncorrelated relaxed lognormal molecular clock and expansion, exponential and constant population growth models. The Expansion model yielded the best results, as indicated by ESS statistics and Bayes factor analysis of the posterior probability trace in TRACER. Sixty million generations were sampled every 1000 states, corresponding to 60 000 trees, that were annotated with TreeAnnotator and visualized with FigTree V1.3.1 from the BEAST package.

## Results and Discussion

In 2008 we received a serum sample for etiological diagnosis of a case of hemorrhagic fever in DRC. The patient' serum was handled under BSL-4 facilities for RNA purification and tested positive for CCHFV by real-time PCR and conventional amplification with previously described detection systems [19], [20]. The patient became ill in Beruwe, approximately 325 km from Kisangani, where the only 2 previously reported cases of CCHFV in DRC occurred in 1956 (Figure 1). The patient worked in a mining area near a forest environment and didn't seem linked to agro pastoral activities. As this was the only identified case of CCHV in DRC for more than 50 years, we performed a phylogenetic analysis to determine whether it was due to introduction of a novel virus or re-emergence of a local genotype.

Virus isolation in Vero cells was unsuccessful, presumably owing to virus degradation subsequently to difficulties and delays of transportation. Genetic characterization was thus based on RT-PCR of RNA extracted from the patient's serum. As reassortment usually affects the M segment, priority was given to sequencing segments S and M, while segment L amplification was limited by sample quantity and was unsuccessful. Near-complete characterization of the segment S coding sequence was achieved, yielding 1501 contiguous nucleotides; the 5′ end was missing, presumably owing to RNA degradation. A 1001-nucleotide fragment was generated for segment M, corresponding to nucleotide positions 2382 to 3380 of the Congo3010-1956 glycoprotein coding sequence (DRC strain).

Pairwise nucleotide comparison of the Beruwe-2008 segment S sequence with those of the most closely related strains Congo3010-1956 (DRC) and Semunya-1958 (Uganda) – showed 92.4% and 92.0% similarity, respectively. In segment M the pairwise identities were 96.1% and 93.8% respectively. Identity between the Beruwe-2008 strain and strains belonging to other genetic groups ranged from 82.2% to 87.6% in segment S and from 72.5% to 81.3% in segment M (Table 2).

**Table 2:** P-distances between the Beruwe-2008 sequences and other sequences included in the phylogenetic analysis.

	P-distance range (%)	
	Segment S	Segment M
**Group 1: West Africa**	[16.1–16.4]	[18.7–19.5]
**Group 2: Central Africa**	[7.6–8]	[3.9–6.2]
**Group 3: South & West Africa**	[14.1–15]	[24.6–27]*
**Group 4: Asia-Middle East**	[12.4–14.8]	
**Group 5: Europe-Turkey**	[13.2–13.8]	[24.4–25.1]
**Group 6: Greece**	[17.8]	[27.5]

*In segment M, phylogeographic groups III and IV are combined and the reported p-distances include both groups.

**Figure 2:**
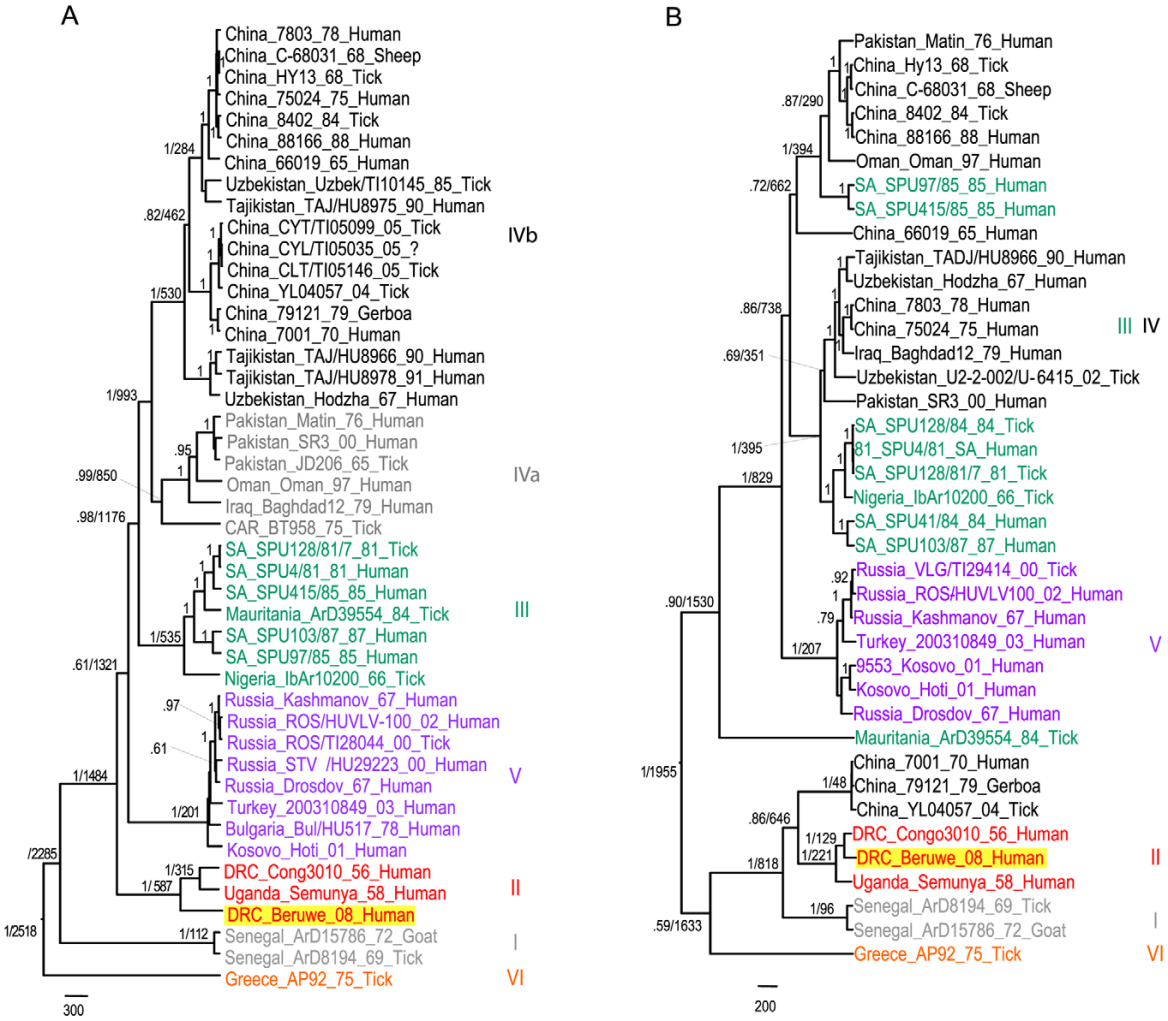
Phylogenetic analysis of CCHFV in segments S and M. Caption A: analysis performed on 1501 nucleotides of segment S (near complete sequence). Caption B: analysis performed on partial segment M sequence, 1001 nucleotides long. Genotypes are indicated in roman numerals, named according to Caroll *et al.* [2] with the equivalent group nomenclature according to Chamberlin et al. [4] indicated in brackets: I – West Africa (Africa 1); II – Central Africa (Africa 2); III – South and West Africa (Africa 3); IV – Middle East/Asia, divided into groups IVa and IVb respectively corresponding to groups Asia 1 and Asia 2 acoording to [4]; V – Europe/Turkey (Europe 1); VI- Greece (Europe 2). The sequence from this study is highlighted in gold. Sequences are named as follows: country_strain_sampling year_host. Posterior probabilities are indicated above branches, followed by node age estimations. For clarity, posterior probability values below 0.6 and date estimates for the terminal branches were removed. The scale bar corresponds to evolutionary distance in years.

Bayesian phylogenetic analysis with a molecular clock assumption was applied to segment S (Figure 2A) and M (Figure 2B) datasets. Both methods yielded tree topologies largely matching the phylogeographic groups previously defined from complete segments S and M [1]–[3]. In both segments, and with posterior probabilities reaching 1, the Beruwe-2008 sequence grouped with the aforementioned DRC and Uganda strains forming lineage II (Central Africa group). Although we cannot rule out the possibility of segment L reassortment, the Beruwe-2008 strain most likely belongs to the genotype previously identified in Central Africa, thus representing viral re-emergence rather than introduction of another genotype. In addition, the phylogenetic position of the Beruwe-2008 strain inside this Central African clade differed between the two segments, lying at the most ancestral branch in segment S while sharing a more recent common ancestry with the Congo3010-1956 strain from Kisangani in segment M. This is highly suggestive of intra-genotypic reassortment, thus implying co-circulation of these two DRC sub-lineages at this time. However, though it may be less probable, we cannot exclude definitively recombination between the two strains.

Our dating analysis of the S segment resulted in time estimations slightly more recent than previously reported, but nonetheless within the same range and in keeping with an ancient origin of CCHF viruses [2]. The MRCA (most recent common ancestor) for the whole CCHFV species was estimated to have arisen 2518 years before present (BP) (95% High Posterior Density (HPD): 820-5281), the lineage II split-off was dated to 1484 years BP (95% HPD: 583-3389) and the MRCA of the three Central African strains was estimated at 587 years BP (95% HPD: 200-1327). In the M segment, the MRCA estimates were slightly more recent, most probably owing to the use of partial rather than complete coding sequences and to different evolution of the two genes. This resulted in MRCA estimates of 1955 years BP for the whole species (95%HPD: 886-3844), 221 years BP for the three Central African strains (95%HPD: 114-407) and 129 years BP (95% HPD: 75-228) for the two DRC strains. The genotype II split-off was estimated to have occurred 646 years BP, but the differences in the tree topologies prevented a true node age comparison with segment S.

CCHV genotype II has been identified only in DRC and Uganda, while different CCHV lineages have been identified in neighboring countries to the north. Multiple genotypes have been identified in CAR, belonging to groups IV and III [2], [20], the latter also being encountered in Sudan [14]. By contrast no other genotype has been identified in Central Africa, for which reports on CCHFV are scarce and date back to 30 years. Hence, the data currently available suggest that genotype II is specific to central Africa. In DRC, CCHV has been reported only once, 50 years ago, but our data strongly suggest that the same genotype is still actively circulating.

Of note, the MRCA estimates presented here are in agreement with ancient divergence of this lineage (around 1000 years ago), but whether or not this split-off was linked to virus adaptation to Central Africa cannot be assessed. However, as the MRCA of the three strains was dated back to 683 to 243 years BP (Figure 2A and B respectively), one might reasonably assume that the association of genotype II with this area goes back to this time period and thus did not result from very recent introduction. In addition, the co-circulation of different sub-lineages supports the possibility that ongoing CCHFV circulation occurred in the same area for some time. However, as the reassortment event would have taken place approximately 120 years BP, there is no evidence that CCHFV has been permanently circulating inside the Beruwe microhabitat, and we cannot exclude the possibility that this virus was very recently (re)introduced.

In addition to the CCHFV genotypic specificity for Central Africa, its occurrence in the tropical rainforest contrasts strongly with the ecological characteristics of other areas in which CCHFV has been isolated [11]. Indeed, the enzootic distribution of CCHFV mostly coincides with temperate to dry or semi-dry climates in the forests, steppes and savannahs of Eurasia and West, East and South Africa. In these environments, domestic animals and their associated ticks are major agents of rural enzootic cycles affecting nearby human populations [11], [25]. Despite the lack of accurate ecological data, the occurrence of CCHFV in Central Africa and its apparent genotypic specificity may suggest a distinctive sylvatic natural cycle in the deep tropical forest characterized by high rainfall, specific wildlife species, and a low density of domestic animals. Interestingly, co-speciation or long-term association with specific tick species has been previously suggested to explain the geographical distribution of CCHV genetic variants in Russia and Central Asia [26]. Such a sylvatic cycle, involving specific vectors and hosts with few contacts with human populations, could partly explain the lack of outbreaks and the sporadic nature of recorded human cases. In addition, as CCHFV is known to have been present in Central Africa for decades, and as human populations often live in isolated villages, many human infections may have been overlooked. However increasing invasion and destruction of rainforest habitats may lead to a higher risk of human CCHFV cases in future.

Hence, despite 30 years without a single reported case, the data presented here suggest that CCHFV continues to circulate in Central Africa. More information on the epidemiology and the natural cycle of CCHFV in this ecosystem is required to assess its potential for emergence, notably in Gabon and Republic of the Congo. However health authorities and medical staff should be aware of the possibility of viral (re)emergence and of the high risk of nosocomial transmission.

## Supporting Information

Table S1 GenBank accession numbers for the sequences used in this study. Countries, strains, date of sampling and hosts are reported along with the associated GenBank accession numbers for segment S and segment M.
(DOC)

## References

[1] Deyde VM, Khristova ML, Rollin PE, Ksiazek TG, Nichol ST (2006). Crimean-Congo hemorrhagic fever virus genomics and global diversity.. J Virol.

[2] Carroll SA, Bird BH, Rollin PE, Nichol ST (2010). Ancient common ancestry of Crimean-Congo hemorrhagic fever virus.. Mol Phylogenet Evol.

[3] Hewson R, Chamberlain J, Mioulet V, Lloyd G, Jamil B (2004). Crimean-Congo haemorrhagic fever virus: sequence analysis of the small RNA segments from a collection of viruses world wide.. Virus Res.

[4] Chamberlain J, Cook N, Lloyd G, Mioulet V, Tolley H (2005). Co-evolutionary patterns of variation in small and large RNA segments of Crimean-Congo hemorrhagic fever virus.. J Gen Virol.

[5] Anagnostou V, Papa A (2009). Evolution of Crimean-Congo Hemorrhagic Fever virus.. Infect Genet Evol.

[6] Hewson R, Gmyl A, Gmyl L, Smirnova SE, Karganova G (2004). Evidence of segment reassortment in Crimean-Congo haemorrhagic fever virus.. J Gen Virol.

[7] Maltezou HC, Andonova L, Andraghetti R, Bouloy M, Ergonul O Crimean-Congo hemorrhagic fever in Europe: current situation calls for preparedness.. Euro Surveill.

[8] Ergonul O (2006). Crimean-Congo haemorrhagic fever.. Lancet Infect Dis.

[9] Swanepoel R, Struthers JK, Shepherd AJ, McGillivray GM, Nel MJ (1983). Crimean-congo hemorrhagic fever in South Africa.. Am J Trop Med Hyg.

[10] Shepherd AJ, Swanepoel R, Shepherd SP, Leman PA, Blackburn NK (1985). A nosocomial outbreak of Crimean-Congo haemorrhagic fever at Tygerberg Hospital. Part V. Virological and serological observations.. S Afr Med J.

[11] Hoogstraal H (1979). The epidemiology of tick-borne Crimean-Congo hemorrhagic fever in Asia, Europe, and Africa.. J Med Entomol.

[12] CRORA viral database. Centre collaborateur OMS de Reference et de Recherche sur les Arbovirus, Institut Pasteur de Dakar.. http://www.pasteur.fr/recherche/banques/CRORA/virus/v0401010.htm.

[13] Nabeth P, Cheikh DO, Lo B, Faye O, Vall IO (2004). Crimean-Congo hemorrhagic fever, Mauritania.. Emerg Infect Dis.

[14] Aradaib IE, Erickson BR, Mustafa ME, Khristova ML, Saeed NS Nosocomial outbreak of Crimean-Congo hemorrhagic fever, Sudan.. Emerg Infect Dis.

[15] Guilherme JM, Gonella-Legall C, Legall F, Nakoume E, Vincent J (1996). Seroprevalence of five arboviruses in Zebu cattle in the Central African Republic.. Trans R Soc Trop Med Hyg.

[16] Degallier N, Cornet JP, Saluzzo JF, Germain M, Herve JP (1985). [Ecology of tick-borne arboviruses in the Central African Republic].. Bull Soc Pathol Exot Filiales.

[17] Simpson DI, Knight EM, Courtois G, Williams MC, Weinbren MP (1967). Congo virus: a hitherto undescribed virus occurring in Africa. I. Human isolations–clinical notes.. East Afr Med J.

[18] Woodall JP, Williams MC, Simpson DI (1967). Congo virus: a hitherto undescribed virus occurring in Africa. II. Identification studies.. East Afr Med J.

[19] Wölfel R, Paweska JT, Petersen N, Grobbelaar AA, Leman PA (2007). Virus detection and monitoring of viral load in Crimean-Congo hemorrhagic fever virus patients.. Emerg Infect Dis.

[20] Rodriguez LL, Maupin GO, Ksiazek TG, Rollin PE, Khan AS (1997). Molecular investigation of a multisource outbreak of Crimean-Congo hemorrhagic fever in the United Arab Emirates.. Am J Trop Med Hyg.

[21] Drummond AJ, Ashton B, Buxton S, Cheung M, Cooper A (2010). Geneious v5.1.. http://www.geneious.com.

[22] Gelman A, Rubin DB (1996). Markov chain Monte Carlo methods in biostatistics.. Stat Methods Med Res.

[23] Ronquist F, Huelsenbeck JP (2003). MrBayes 3: Bayesian phylogenetic inference under mixed models.. Bioinformatics.

[24] Drummond AJ, Rambaut A (2007). BEAST: Bayesian evolutionary analysis by sampling trees.. BMC Evol Biol.

[25] Whitehouse CA (2004). Crimean-Congo hemorrhagic fever.. Antiviral Res.

[26] Yashina L, Petrova I, Seregin S, Vyshemirskii O, Lvov D (2003). Genetic variability of Crimean-Congo haemorrhagic fever virus in Russia and Central Asia.. J Gen Virol.

