# Supplementary material for: Re-Emergence of Crimean-Congo Hemorrhagic Fever Virus in Central Africa
Source: PLoS Negl Trop Dis. 2011 Oct 11;5(10):e1350. doi: 10.1371/journal.pntd.0001350 (PMC3191127; doi:10.1371/journal.pntd.0001350)
Supplement: Table S1 — GenBank accession numbers for the sequences used in this study. Countries, strains, date of sampling and hosts are reported along with the associated GenBank accession numbers for segment S and segment M. (DOC) [file pntd.0001350.s001.doc]

**Table S1.** GenBank accession numbers for the sequences used in this study.

**Countries, strains, date of sampling and hosts are reported along with the associated GenBank accession numbers for segment S and segment M.**

| **Country** | **Strain** | **Year** | **Host** | **Segment S** | **Segment M** |
| --- | --- | --- | --- | --- | --- |
| **Bulgaria** | Bul/HU517 | 1978 | Human | AY277676 |  |
| **CAR** | BT958 | 1975 | Tick | EF123122 |  |
| **China** | 66019 | 1965 | Human | AJ010648 | AB069669 |
| **China** | C-68031 | 1968 | Sheep | DQ211642 | DQ211629 |
| **China** | HY13 | 1968 | Tick | CHU88413 | AY900145 |
| **China** | 7001 | 1970 | Human | AF415236 | AB069670 |
| **China** | 75024 | 1975 | Human | AF362080 | AB069671 |
| **China** | 7803 | 1978 | Human | AF354296 | AB069672 |
| **China** | 79121 | 1979 | Gerboa | AF358784 | AB069673 |
| **China** | 8402 | 1984 | Tick | AJ010649 | AB069674 |
| **China** | 88166 | 1988 | Human | AY029157 | AB069675 |
| **China** | YL04057 | 2004 | Tick | FJ562093 | FJ562094 |
| **China** | CLT/TI05146 | 2005 | Tick | DQ227496 |  |
| **China** | CYL/TI05035 | 2005 |  | DQ217602 |  |
| **China** | CYT/TI05099 | 2005 | Tick | DQ227495 |  |
| **DRC** | Congo 3010 | 1956 | Human | DQ144418 | DQ211637 |
| **DRC** | **Beruwe-2008** | **2008** | **Human** | **HQ849545** | **HQ849546** |
| **Greece** | AP92 | 1975 | Tick | DQ211638 | DQ211625 |
| **Iraq** | Baghdad12 | 1979 | Human | AJ538196 | AJ538197 |
| **Kosovo** | 9553 | 2001 | human |  | AY675511 |
| **Kosovo** | Hoti | 2001 | Human | DQ133507 | EU037902 |
| **Mauritania** | ArD39554 | 1984 | Tick | DQ211641 | DQ211628 |
| **Nigeria** | IbAr10200 | 1966 | Tick | CHU88410 | AF467768 |
| **Oman** | Oman | 1997 | Human | DQ211645 | DQ211632 |
| **Pakistan** | JD206 | 1965 | Tick | CHU88414 |  |
| **Pakistan** | Matin | 1976 | Human | AF527810 | AF467769 |
| **Pakistan** | SR3 | 2000 | Human | AJ538198 | AJ538199 |
| **Russia** | Drosdov | 1967 | Human | DQ211643 | DQ211630 |
| **Russia** | Kashmanov | 1967 | Human | DQ211644 | DQ211631 |
| **Russia** | ROS/TI28044 | 2000 | Tick | AY277672 |  |
| **Russia** | STV/HU29223 | 2000 | Human | AF481802 |  |
| **Russia** | VLG/TI29414 | 2000 | Tick |  | AY179961 |
| **Russia** | ROS/HUVLV-100 | 2002 | Human | DQ206447 | DQ206448 |
| **SA** | SPU128/81/7 | 1981 | Tick | DQ076415 | DQ157174 |
| **SA** | SPU4/81 | 1981 | Human | DQ076416 | DQ157175 |
| **SA** | SPU128/84 | 1984 | Tick |  | AY900141 |
| **SA** | SPU41/84 | 1984 | Human |  | AY900142 |
| **SA** | SPU415/85 | 1985 | Human | DQ211648 | DQ211635 |
| **SA** | SPU97/85 | 1985 | Human | DQ211646 | DQ211633 |
| **SA** | SPU103/87 | 1987 | Human | DQ211647 | DQ211634 |
| **Senegal** | ArD8194 | 1969 | Tick | DQ211639 | DQ211626 |
| **Senegal** | ArD15786 | 1972 | Goat | DQ211640 | DQ211627 |
| **Tajikistan** | TAJ/HU8966 | 1990 | Human | AY049083 | AY179962 |
| **Tajikistan** | TAJ/HU8975 | 1990 | Human | AY297692 |  |
| **Tajikistan** | TAJ/HU8978 | 1991 | Human | AY297691 |  |
| **Turkey** | 200310849 | 2003 | Human | DQ211649 | DQ211636 |
| **Uganda** | Semunya | 1958 | Human | DQ076413 | DQ094832 |
| **Uzbekistan** | Hodzha | 1967 | Human | AY223475 | AY223476 |
| **Uzbekistan** | Uzbek/TI10145 | 1985 | Tick | AF481799 |  |
| **Uzbekistan** | U2-2-002/U-6415 | 2002 | Tick |  | AY900144 |

CAR: Central African Republic, SA: South Africa.
